# Smoking and prostate cancer: a life course analysis

**DOI:** 10.1186/s12885-018-4065-7

**Published:** 2018-02-07

**Authors:** Evelyn Jiménez-Mendoza, Ruth A. Vázquez-Salas, Tonatiuh Barrientos-Gutierrez, Luz Myriam Reynales-Shigematsu, Isaac Roberto Labra-Salgado, Hugo A. Manzanilla-García, Luisa E. Torres-Sánchez

**Affiliations:** 1 Instituto Nacional de Salud Pública (INSP), Av. Universidad 655, Col. Sta. María Ahuacatitlán, 62100 Cuernavaca, Morelos México; 2 Hospital General de México, Dr. Balmis 148, Col. Doctores, Deleg. Cuauhtémoc, 06726 México, Ciudad de México Mexico

**Keywords:** Gleason, Prostate cancer, Smoking index, Smoking patterns, Mexico

## Abstract

**Background:**

Inconsistent associations between smoking status and prostate cancer (PC) could be due to exposure assessment error. Reconstructing smoking behaviors over the life course could reduce exposure assessment error.

**Methods:**

As part of a case-control study, we identified 402 incident and histologically confirmed PC cases that were matched by age (±5 years) to 805 population controls. Through direct interview, we obtained information about: age at smoking onset, intensity and frequency of cigarette smoking at different life stages, and smoking cessation age. Smoking status at interview and average smoking index over the lifetime (packs/year) were estimated. Life course smoking patterns were obtained applying the k-means+ method for longitudinal data to the smoking index (pack/year) for each life stage.

**Results:**

Two life-course smoking patterns were identified among ever smokers: “pattern A” characterized by males who reported low and constant smoking intensity (87.8%), and “pattern B” (12.2%) males with an initial period of low intensity, followed by an increase during the second period. Compared to never smokers, pattern B was associated with higher poorly differentiated PC, (OR 2.30; 95% CI 1.21–4.38). No association was observed with average smoking index.

**Conclusion:**

Life course smoking patterns seem to capture the smoking variability during life course and reduce the likelihood of reverse causation. Using this assessment strategy our findings support the potential role of tobacco smoking in PC, particularly poorly differentiated PC. Prospective studies with comprehensive smoking history during the lifetime are needed to confirm these findings.

**Electronic supplementary material:**

The online version of this article (10.1186/s12885-018-4065-7) contains supplementary material, which is available to authorized users.

## Background

Tobacco smoking is a primary cause of various cancers [1]. However, studies analyzing the association between tobacco and prostate cancer (PC) have found mixed results. A recent meta-analysis with prospective studies [2], found a modest but statistically significant association between cigarette smoking and fatal PC. Important sources of heterogeneity included study completion before the prostate specific antigen-screening era, lack of PC aggressiveness consideration and differences in the smoking assessment methods [2].

Smoking assessment is a major challenge in prostate cancer studies. Some studies use smoking status at interview [3–10], age at smoking onset [10, 11], total consumption time [5–7, 11], number of cigarettes [3, 6–10], years since cessation [6, 10, 12] or smoking index (pack/years) [4–8, 11–13]. The smoking index (pack/years) at different life stages or across the lifespan is considered the gold standard for smoking assessment; however, PC studies that use the smoking index report inconsistent results [4, 5, 7, 8, 11, 13]. Reasons for inconsistency are related to various factors. For instance, the smoking index over the lifespan averages packs/year for the duration of life, eliminates information that could be relevant, such as the variability in frequency, duration and intensity that occur throughout life. Thus, it is necessary to develop new approaches to smoking assessment in PC studies, allowing for analyses of smoking patterns over time.

To date, no retrospective study has taken a life course approach to assess the association between smoking and prostate cancer. We aimed to evaluate the association between prostate cancer and life course smoking history in a case-control study [14] carried out in Mexico, where PC screening is low and a high proportion of PC cases at diagnosis are classified as aggressive [15]. To achieve this goal we reconstructed the life course smoking history throughout life using the smoking index at different life stages, assessing its association with PC and PC aggressiveness.

## Methods

From November 2011 to August 2014, we conducted a case-control study about PC risk factors with men aged 42–94 years; study details were reported previously [14]. Briefly, cases were males with incident and histologically confirmed PC diagnosis, identified at two secondary [Hospital General Regional No. 1 “Dr. Carlos McGregor Sánchez Navarro” (IMSS), and Hospital Regional “Adolfo López Mateos” (ISSSTE)] and four tertiary level hospitals [(Hospital General de México (SSA), Instituto Nacional de Cancerología (SSA), Instituto de Ciencias Médicas y Nutrición Salvador Zubirán (SSA), Hospital de Oncología del Centro Médico Siglo XXI (IMSS)]. All cases were residents of Mexico City for at least 1 year and had no previous history of cancer. We categorized PC cases according to Gleason score at diagnosis into less (< 7) or highly aggressive (≥7). Also, using the National Comprehensive Cancer Network classification, we categorized PC cases as: well differentiated (Gleason ≤6), moderately differentiated (Gleason 7), or poorly differentiated (Gleason ≥8) [16]. All cases were interviewed at the hospital, before they knew their definitive diagnosis.

For each case, two controls (2:1) caliper-matched by age (±5 years) were selected in the community; inclusion criteria for time residing in Mexico City and history of cancer were the same as for cases. Subjects were not eligible to become controls if they experienced urological symptoms (dysuria, hematuria), have had a clinical evaluation for prostate disease, or had a previous Prostate Specific Antigen (PSA) ≥4 ng/mL. Controls were recruited from 33 basic geostatistical areas selected using probability proportional to number of households as reported by National Institute of Statistics and Geography (INEGI, acronym in Spanish). Participating households were randomly selected and a first visit was scheduled to verify how many males met the inclusion criteria. If more than one male was eligible, we randomly selected one to participate in the study. If a potential control was not present at home, we made up to three attempts to locate him, before selecting another control. All controls were interviewed at their home.

Cases and controls who refused to participate in the study answered a short questionnaire about sociodemographic characteristics (age, birthplace, marital status and educational level).

### Interview

Through a face-to-face interview and using a structured questionnaire, trained staff unaware of the specific study hypothesis obtained information from each participant. This information included: sociodemographic characteristics, family history of cancer in first-degree relatives (prostate, breast, ovary and colon cancer), personal history of chronic diseases (diabetes, dyslipidemias, hypertension, and others), history of sexually transmitted diseases, smoking, physical activity (PA), and diet. The average duration of the interview was 45 min.

### Smoking history

Smokers were identified using the question “Have you ever smoked 100 cigarettes in your life?” Smokers were further asked questions (see Additional file 1) about their smoking history, including: age at smoking onset and age of smoking cessation, current smoking status and number of cigarettes smoked per day at interview and at different life stages (≤20, 21–30, ≥ 31 years old).

Based on the smoking status at interview we categorized participants as never, former or current smokers. The cumulative lifespan smoking index was estimated multiplying the average number of cigarettes by the number of smoking years and divided by 20 [17]. This index was expressed as smoked packs per year and was categorized according to the tertile distribution of ever smokers among the controls (≤5.2, 5.3–14.0 and ≥14.01packs/year). Finally, we constructed the smoking index (packs/year) for each of the following life stages: ≤ 20, 21–30, ≥ 31 years.

For the life course analysis of smoking among ever and former smokers, we considered the smoking index (pack/year) from each life stage and used the k-means+ method [18] for longitudinal data in R software to extract the smoking patterns (see Additional file 2). Using this method instead of establishing the instantaneous smoking status at the interview, we attempted to document the full smoking history. All patterns were verified by the quality criteria of Calinski & Harabasz; moreover, we reconstructed the smoking patter by k-means++ method (Additional file 2) and obtained similar patterns. The reference category for all analyses was never smokers. To provide a description of current smoking and the average smoking index, we cross-tabulated these variables with the life-time smoking patterns.

### Diet

Dietary information was obtained by a previously validated food frequency questionnaire [19] that includes 127 food-groups and took as timeframe 3 years before diagnosis for cases and 3 years before interview for controls. The frequency intake ranged from 0 to 6 times per day; frequencies were transformed to daily intake. The dairy intake group was constructed using cheese (Oaxaca, freshly made and Manchego) and yogurt consumption. Total energy intake was calculated using the food composition database from the Food Processor Nutrition Analysis and Fitness Software (version 10.11.0, 2011, ESHA Research Inc., Salem, OR), which includes data on Mexican food composition. Those energy intake values less than 800 or more than 4500 cal/d, were considered as extreme values and excluded from the analysis.

### Physical activity

Using leisure-time physical activity (PA) information for three different life stages, we estimated the total energy expenditure in metabolic equivalents (Met/min/day/year) and reconstructed life course PA patterns. Among males who reported any leisure-time PA, we identified three patterns: Pattern A, males who had high PA intensity at 15–18 years and decreased over their life course; Pattern B, males who maintained a constantly low PA intensity; and Pattern C, males who constantly performed more PA.

### Statistical analysis

Smoking history and participant characteristics were compared between cases and controls; according to the variable type we employed *t-Student* or χ^2^ for continuous or categorical variables, respectively. Among controls the median age at smoking onset, smoking intensity (average cigarettes per day), and smoking duration in years were compared across smoking assessment approaches, using the Wilcoxon rank-sum test*.*

To analyze the association between life course smoking patterns with PC we used unconditional logistic regression models. To estimate the association between PC aggressiveness and life course smoking patterns, we compared all controls to cases with well-differentiated (Gleason ≤6), moderately differentiated (Gleason 7), or poorly differentiated (Gleason ≥8) cancer. Furthermore, we evaluated the association between other smoking approaches (smoking status at interview, and the average smoking index during the lifetime) and PC, as well as PC aggressiveness.

All models were adjusted by age at interview. As potential confounders, we selected variables that, according to the literature, are known risk factors for the association between smoking and PC. Besides age at interview, final models included only variables changing the crude estimator by > 10%: educational level, family history of prostate cancer in first-degree relatives, chronic and sexually transmitted diseases, life course of physical activity, dairy and energy intake. Models for smoking status at interview were also adjusted by number of cigarettes and smoking duration.

Because previous studies have differentiated former smokers by their abstinence time [6, 12, 13], we evaluated the association between smoking status with PC and PC aggressiveness using three cut-off points of abstinence (10, 15 and 20 years) for former smokers. Finally, we evaluated the possibility and magnitude of an exposure measurement error, considering different smoking assessment approaches. Among controls, we compared the proportion of subjects classified by smoking index and smoking status with the observed life course smoking patterns.

Analyses were performed in Stata ver. 13.0 and R Studio ver. 3.0.2.

## Results

From 468 eligible cases and 920 eligible controls, 402 (85.9%) cases and 805 controls (87.5%) accepted to participate; the main decline reason was lack of interest. Additionally, 8 cases and 11 controls were excluded from the analysis because they had out of range total energy values. Our final sample size was 394 cases and 794 controls. Regarding PC aggressiveness, Gleason information was available for 383 cases (95.3%); of them, 26.4% were Gleason < 7, 37.3% were Gleason 7 and 35.7% were Gleason ≥8.

Table 1 presents selected characteristics of cases and controls. Compared to controls, cases had higher educational attainment (university or more 20.6 vs. 11.7%), history of chronic diseases (58.1 vs. 41.2%), history of sexually transmitted diseases (26.7 vs. 11.0%), and family history of PC (10.2 vs. 2.6%). Dairy consumption (41.8 vs. 32.2%) and energy intake (2172.13 ± 717.81 vs. 1959.28 ± 681.88) was also higher among cases than controls. In contrast, a higher proportion of controls than cases (73.7 vs. 60.4%) reported constant life course physical activity (patterns B and C) (Table 1).

**Table 1:** Selected characteristics of the study population according to cases and controls

Characteristics	Cases (*n* = 394)	Controls (*n* = 794)	*p* value^a^
Marital status (%)			
United^b^	304 (77.2%)	636 (80.1%)	0.24
Not united	90 (22.8%)	158 (19.9%)	
Educational level (%)			
Elementary school or less	177 (44.9%)	358 (45.1%)	< 0.001
Junior high school	66 (16.7%)	199 (25.1%)	
High school	70 (17.8%)	144 (18.1%)	
University or more	81 (20.6%)	93 (11.7%)	
History of chronic diseases^c^ (%)			
Yes	229 (58.1%)	327 (41.2%)	< 0.001
No	165 (41.9%)	467 (58.8%)	
History of STD^d^ (%)			
Yes	105 (26.7%)	87 (11.0%)	< 0.001
No	288 (73.1%)	706 (88.9%)	
Missing	1 (0.2%)	1 (0.1%)	
Family history of PC^e^ (%)			
Yes	41 (10.2%)	20 (2.6%)	< 0.001
No	353 (89.6%)	774 (97.5%)	
Dairy consumption (portion/d) (%)			
Low	124 (32.1%)	286 (36.7%)	0.004
Middle	104 (26.1%)	251 (31.1%)	
High	166 (41.8%)	257 (32.2%)	
Energy			
Mean ± SD	2172.13 ± 717.81	1959.28 ± 681.88	< 0.001
Life course PA patterns^f^ (%)			
None	58 (14.7%)	74 (9.3%)	< 0.001
A	98 (24.9%)	135 (17.0%)	
B	218 (55.3%)	532 (67.0%)	
C	20 (5.1%)	53(6.7%)	

^a^t-test or χ^2^ test

^b^United: Married and common law

^c^History of hypertension, diabetes and/or dyslipidemia

^d^History of sexual transmitted diseases

^e^Family history of PC in first degree relatives

^f^Pattern A, males who had high PA intensity at 15-18 years old and decreased over their life course; Pattern B, males who maintained a constantly low PA intensity and Pattern C, males with constantly high PA intensity

**Fig. 1:**
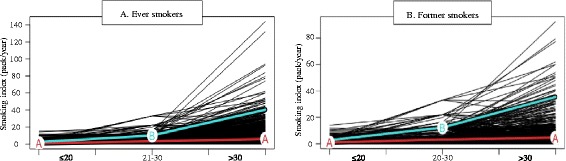
Life course smoking patterns among ever (A) and former (B) smokers. a. Among ever smokers: Pattern A, characterized by males who reported low and constant smoking intensity (87.8%) and Pattern B, males with an initial period of low smoking intensity, followed by an increase during the second period (12.2%). b. Among former smokers: Pattern A, characterized by males who reported a low and constant smoking intensity (88.7%) and Pattern B, males with an initial period of low smoking status, followed by an increase during the second period (11.3%)

Approximately 67% of men in the sample reported a history of tobacco-use and most of them were former smoker at interview (63.8%). Regarding the average smoking intensity during the lifetime only 35.5% reported 14 or more packs/year. Through life course analysis among ever smokers, we identified two smoking patterns (Fig. 1a): pattern A (87.8%) characterized by a smoking intensity ranged between 0.93 to 5.92 pack per year at the stages ≤20 years old and ≥30 years old, respectively. In contrast, subjects in pattern B (12.2%) began with an average smoking intensity of 2.5 pack per year at ≤20 years old stage, followed by an increase starting in the 21–30 years old period (10.2 pack/year), reaching an average 39.5 pack per year at ≥30 years old stage. Among former smokers (Fig. 1b), we identified similar patterns; however, pattern B (11.3%) had a higher smoking intensity starting at 21–30-year-old period (12.25 vs. 10.2 pack/year) and reaching 35.4 pack per year at last stage (Additional file 3).

Comparisons between life course smoking patterns, current smoking status and average smoking index in terms of key smoking parameters among controls are presented in Table 2. Differences in age at smoking onset and number of cigarettes were smaller across categories of smoking status at interview than across tertiles of average smoking index or between patterns A and B of life course smoking. Median smoking duration ranged from 47 years in current smokers to 26 years in former smokers; similar to that observed between the lowest and highest tertile of the average smoking index (23 to 45 years). In contrast, smoking pattern A had an average smoking duration of 35 years, compared to 48 years in pattern B.

**Table 2:** Age at smoking onset, intensity, and smoking duration among controls according to selected smoking approaches

Smoking assessment approaches	n (%)	Age at smoking onset (years) Median (Min-Max)	Number of cigarettes/day Median (Min-Max)	Duration (years) Median (Min-Max)
Smoking status at interview				
Never	261 (32.9)	–	–	–
Former	301 (37.9)	17 (6–55)	4.7 (1.0–60)	26 (2–64)
Current	232 (29.2)	17 (8–53)	5.0 (0.7–40.0)	47 (15–74)^a^
Average smoking index during the life time (packs/year)				
0.15–5.2	177 (33.2)	18 (8–55)	2.0 (0.7–20.0)	23 (2–59)
5.3–14.0	179 (33.6)	17 (7–40)	4.3 (2.0–30)	40 (6–69)
14.01–112.0	177 (33.2)	15 (6–40)^a^	11.6 (5.0–60.0)^a^	45 (9–74)^a^
Life course smoking patterns				
Among ever smokers^b^				
A	474(88.9)	17 (7–55)	4 (0.7–40) 15.5	35 (2–69)
B	59 (11.1)	15 (6–25)	(5.75–60.0)^a^	48 (20–74)^a^
Among former smokers^b^				
A	277 (92.0)	17 (7–55)	4.3 (1–30)	25 (2–64)
B	24 (8.0)	15 (6–18)^a^	17.9 (8.7–60)^a^	42.5 (20–56)^a^

^a^Wilcoxon rank-sum test *p*<0.05

^b^Pattern A, characterized by males who reported low and constant smoking intensity and Pattern B, males with initial period of low smoking intensity, followed by an increase during the second period

Association between smoking assessment approaches and prostate cancer are show in Table 3. In age-adjusted models, current smokers had 50% lower odds of PC than never smokers (OR 0.5; 95%CI 0.35, 0.72) while former smokers had 41% higher odds of PC compared to never smokers (OR 1.41; 95% CI 1.07, 1.86). In fully adjusted models, only the association between current smokers and PC remained (OR 0.42; 95%CI 0.20, 0.86). No associations between PC and average lifespan smoking index (pack/year) were observed. Life course smoking patterns among ever smokers were not associated with PC (Table 3). However, when we used the life course smoking patterns estimated among former smokers, males in Pattern B had a higher probability of PC (OR 2.49; 95% CI 1.35–4.58) than never smokers.

**Table 3:** Association between smoking history and prostate cancer, using different smoking assessment approaches

Smoking assessment approaches.	Cases n = 394 (%)	Controls n = 794 (%)	OR^a^	95% CI	OR^b^	95% CI
Smoking status at interview						
Never	128(32.5)	261(32.9)	1.0	–	1.0	–
Former	209(53.0)	301(37.9)	1.41	1.07–1.86	1.13	0.70–1.83
Current	57(14.5)	232(29.2)	**0.50**	**0.35–0.72**	**0.42**	**0.20–0.86**
Average smoking index in life (pack/year)						
Never smoker	128(32.5)	261(32.9)	1.0	–	1.0	–
0.15–5.2	85(21.6)	177(22.3)	0.98	0.70–1.38	1.07	0.75–1.53
5.3–14.0	74 (18.8)	179 (22.5)	0.84	0.60–1.18	0.90	0.62–1.30
14.01–112.0	107 (27.2)	177(22.3)	1.22	0.89–1.68	1.13	0.80–1.60
Life course smoking patterns						
Among ever smoker^c^						
Never smoker	128(32.5)	261(32.9)	1.0	–	1.0	–
A	228(57.9)	474(59.7)	0.99	0.77–1.29	1.06	0.80–1.41
B	38(9.6)	59(7.4)	1.30	0.82–2.05	1.15	0.70–1.89
Among former smoker^c^						
Never smoker	128(38.0)	261(46.4)	1.0	–	1.0	–
A	175(51.9)	277(49.3)	1.27	0.96–1.69	1.32	0.97–1.79
B	34(10.1)	24(4.3)	**2.85**	**1.62–5.01**	**2.49**	**1.35–4.58**

^a^Adjusted by age at interview

^b^Adjusted by age at interview, educational level, family history of prostate cancer in first-degree relatives, chronic and sexually transmitted diseases, life course physical activity, dairy and energy intake. Smoker status at interview model, also was adjusted by number of cigarettes and smoking duration

^c^Pattern A: characterized by males who reported low and constant smoking intensity, Pattern B: males with an initial period of low smoking intensity, followed by an increase during the second period

Text in bold denotes statistical significance

Table 4 shows the association between smoking history and PC aggressiveness at diagnosis. Like previous findings, being a current smoker at interview was only associated with lower moderately differentiated PC (Gleason 7). No associations between PC and average smoking index were observed. Furthermore, for life course smoking patterns among ever smokers, pattern B was associated with poorly differentiated PC (OR _Gleason ≥ 8_ 2.30; 95% CI 1.21–4.38) and no association was observed with pattern A. Similarly, the odds of highly aggressive PC were higher in former smokers classified as pattern B (OR _Gleason ≥ 7_ 2.59; 95% CI 1.35–4.98; OR _Gleason ≥ 8_ 4.65; 95% CI 2.17–9.92).

**Table 4:** Association between smoking history and prostate cancer aggressiveness at diagnosis

Smoking assessment approaches.	Gleason ≤ 6		Gleason ≥ 7		Gleason = 7		Gleason ≥ 8	
	Cases *n* = 101	OR (95% CI)	Cases *n* = 282	OR (95% CI)	Cases *n* = 140	OR (95% CI)	Cases *n* = 134	OR (95% CI)
Smoking status at interview^a^								
Never	35	1.0	91	1.0	47	1.0	39	1.0
Former	54	1.30(0.58–2.85)	147	1.03(0.60–1.77)	73	1.03(0.51–2.07)	72	0.99 (0.47–2.08)
Current	12	0.57(0.12–2.80)	44	0.97(0.35–2.68)	20	**0.33(0.11–0.93)**	23	0.42 (0.34–5.35)
Average smoking index in life (packs/years)^b^								
Never smoker	35	1.0	91	1.0	47	1.0	39	1.0
0.15–5.2	24	1.08(0.61–1.94	57	1.03(0.69–1.55)	29	0.98(0.58–1.65)	27	1.15 (0.65–2.0)
5.3–14.0	16	0.73(0.38–1.39)	57	0.98(0.66–1.48)	29	1.02(0.61–1.72)	27	1.06 (0.60–1.86)
14.01–112.0	26	1.08(0.61–1.90)	77	1.14(0.78–1.67)	35	1.04(0.63–1.72)	41	1.42 (0.85–2.36)
Life course smoking patterns								
Among ever smoker^b, c^								
Never	35	1.0	91	1.0	47	1.0	39	1.0
A	59	1.02(0.64–1.63)	160	1.05(0.76–1.45)	84	1.36(0.88–2.11)	73	1.11(0.71–1.74)
B	7	0.85(0.35–2.08)	31	1.30(0.76–2.22)	9	1.64(0.65–4.15)	22	**2.30 (1.21–4.38)**
Life course smoking patterns								
Among former smoker^b, c^								
Never	35	1.0	91	1.0	47	1.0	39	1.0
A	46	1.29(0.78–2.13)	121	1.30 (0.92–1.83)	65	1.35 (0.64–2.84)	54	1.34 (0.83–2.17)
B	8	2.58(0.99–6.75)	26	**2.59 (1.35–4.98)**	8	3.23 (0.64–16.3)	18	**4.65 (2.17–9.92)**

Text in bold denotes statistical significance

Gleason ≤6 well-differentiated PC, Gleason ≥7 highly aggressive PC, Gleason = 7 Moderately differentiated PC and Gleason ≥8 Poorly differentiated

Gleason information was available for 383 cases

^a^All models for smoker status at interview, were adjusted by age at interview, educational level, family history of prostate cancer in first-degree relatives, chronic and sexually transmitted diseases, life course physical activity, dairy and energy intake, number of cigarettes and smoking duration

^b^All models were adjusted by age at interview, educational level, family history of prostate cancer in first-degree relatives, chronic and sexually transmitted diseases, life course physical activity, dairy and energy intake

^c^Pattern A: characterized by males who reported low and constant smoking intensity, Pattern B: males with an initial period of low smoking intensity, followed by an increase during the second period

Table 5 shows the association between smoking status and PC as well as PC aggressiveness, considering the cessation time at interview for former smokers. Compared to never smokers, former smokers who stopped smoking 15 years ago and more was not associated with PC. Meanwhile, among those with a cessation time shorter than 15 years before the interview the odds of PC were higher (OR _All_ 3.33; 95% CI 1.47–7.58) as well as for aggressive PC (OR _Gleason ≥ 8_ 3.81; 95% CI 1.10–13.1). Similar results were observed when we used the 20 years cut-off point.

**Table 5:** Smoker status and its association with prostate cancer and aggressiveness taking into account cessation time at interview

Smoking status at interview.	Controls *n* = 794	Overall PC			Gleason < 7			Gleason = 7			Gleason ≥ 8		
		Cases *n* = 394	OR^a^	95% CI	N	OR^a^	95% CI	n	OR^a^	95% CI	n	OR^a^	95% CI
Never	261	128	1.0	–	35	1.0	–	47	1.0	–	39	1.0	–
Former	301	209	1.13	0.70–1.83	54	1.30	0.58–2.85	73	1.03	0.51–2.07	72	0.99	0.47–2.08
Current	232	57	0.42	0.20–0.86	12	0.57	0.12–2.80	20	0.33	0.11–0.93	23	0.42	0.34–5.35
Never	261	128	1.0	–	35	1.0	–	47	1.0	–	39	1.0	–
Former^b^													
> 10 years	259	147	1.40	0.86–2.30	35	2.51	0.62–10.1	54	1.17	0.57–2.38	52	1.21	0.57–2.59
≤ 10 years	42	62	**4.44**	**1.90–10.4**	19	1.65	0.73–3.72	19	2.25	0.67–7.54	20	3.42	0.96–12.2
Current	232	57	0.95	0.41–2.19	12	1.80	0.40–8.20	20	0.52	0.16–1.76	23	0.88	0.25–3.09
Never	261	128	1.0	–	35	1.0	–	47	1.0	–	39	1.0	–
Former^b^													
> 15 years	204	114	1.36	0.83–2.24	30	1.38	0.61–3.15	41	1.13	0.55–2.30	39	1.28	0.59–2.75
≤ 15 years	97	95	**3.33**	**1.47–7.58**	24	2.64	0.68–10.5	32	1.72	0.54–5.49	33	**3.81**	**1.10–13.2**
Current	232	57	1.05	0.42–2.62	12	0.57	0.12–2.80	20	0.51	0.14–1.94	23	1.34	0.34–5.35
Never	261	128	1.0	–	35	1.0	–	47	1.0	–	39	1.0	–
Former^b^													
> 20 years	169	91	1.28	0.78–2.08	23	1.44	0.64–3.24	33	1.08	0.54–2.21	31	1.18	0.55–2.54
≤ 20 years	132	118	**3.22**	**1.45–7.11**	31	3.44	0.94–12.6	40	1.68	0.55–5.16	41	**3.78**	**1.14–12.5**
Current	232	57	1.15	0.45–2.95	12	0.93	0.18–4.62	20	0.53	0.14–2.07	23	1.55	0.37–6.43

Gleason information was available for 383 cases; 8 cases were not considered in stratified analyses Gleason = 7 or Gleason ≥8, because they were just classified as Gleason ≥7

Text in bold denotes statistical significance

^a^Adjusted by age at interview, educational level, family history of prostate cancer in first-degree relatives, chronic and sexually transmitted diseases, life course physical activity, dairy energy intake and number of cigarettes and smoking duration

^b^Former smokers were categorized according to cessation time before interview

## Discussion

We aimed to analyze the association between PC and current smoking status, average smoking index over the lifetime and life course patterns of smoking behaviors. Current smokers experienced lower odds of PC and PC aggressiveness than never smokers; no association between PC and former smoker was observed. No association between average smoking index over the lifetime (pack/years) and PC or PC aggressiveness was observed. Among ever smokers, having a life course smoking pattern that intensified the smoking frequency (pattern B) was associated with a greater possibility of PC, mainly at the expense of poorly differentiated PC. Among former smokers, pattern B also was associated with higher odds of PC and PC aggressiveness.

There is sufficient evidence to consider tobacco smoking as a potential cause of prostate cancer. PC is a hormone-dependent cancer and alterations on sexual hormonal bioavailability caused by smoking have been described [20, 21]. Cigarette smoking is associated with a significant serum level increase of testosterone, sexual hormone binding globulin (SHBG) [6, 22], as well as estradiol bioavailability [23]. There is evidence that testosterone and estradiol seems to be involved in prostatic cell promotion and tumor growth mechanism [24, 25]. Another potential mechanism is related to the multiple carcinogenic compounds present in cigarettes, most of them (aldehydes, benzene, metals -cadmium, arsenic, beryllium and lead-, nitrosamines and PAHs) capable of producing cell proliferation, genotoxicity and inflammation [26]. Considering the plausibility of the involvement of smoking in PC, inconsistent associations found in the literature could be attributed to errors in exposure assessment.

PC studies have frequently used smoking status at interview as the main exposure variable. In our analysis smokers at interview experienced lower odds of PC than never smokers, a finding that has been reported in some studies using smoking status at interview [2, 11]. Likely, this finding responds to the increase in smoking cessation that occurs when people are diagnosed with PC or other chronic disease [27, 28]. Thus, using smoking status at the time of the interview is subject to information bias and should be discouraged. Also, we did not observe an association between former smoking at interview and PC; this is likely due to the mix of recent and long-standing former smokers, as well as to the proportion of current smokers who misclassify themselves into former-smokers due to the pressure to quit smoking [29]. This mixture should lead the association towards the null under the assumption that long-term former smokers have less PC than men who remained smoking for a longer period of time. Former smoking status and cessation time seems to be informative to PC; however, cut-off points are arbitrary, and previous studies have chosen points ranging from current to 10 years without providing a clear rationale for using either one. Life course analysis presents an important advantage over former smoking cut-offs, as it defines smoking patterns based on actual information about duration and smoking intensity, eliminating the arbitrariness of the process.

The smoking index is considered the gold standard for smoking exposure assessment. In our study we failed to observe an association between the smoking index and PC as well as PC aggressiveness. Our findings are similar to those reported in the literature [4, 5, 7, 8, 11, 13], although other authors have found an association [6, 12]. The smoking index is built on the premise that smoking remains constant over time, as it averages smoking intensities across life stages; however, it is a poor method to capture changes in smoking intensity, and as such it could introduce misclassification. In our study, all controls classified as light and middle smokers by the smoking index belonged to pattern A of smoking. However, only 33% of subjects classified as heavy smokers increased their smoking intensity from early adulthood (pattern B), while the rest maintained constant levels of smoking (Additional file 4). Constant heavy smokers, as classified with the smoking index, showed no association with PC, but Pattern B increased the odds of PC; this finding suggests that capturing increases in the intensity of smoking could be more relevant to PC than estimating the average consumption level over the lifetime. Confirmation of these findings by other studies is needed, but they open the possibility to identify critical windows of exposure to develop PC.

Our study is unique due to the use of life course smoking patterns. To the best of our knowledge, only a study by Giovannucci et al., [6], used a similar approach and their results are consistent with ours. In the context of a prospective cohort study, they estimated the smoking index at baseline and updated it every 2 years. They found a significant increase in metastatic and fatal PC risk for each increment of 15 pack-years of cigarettes smoked (p for trend≤0.03) in the prior decade before PC diagnosis. Unfortunately, no other studies have used a life course approach to capture the complexity of smoking behaviors. The evaluation of life course smoking patterns could provide a more comprehensive characterization of smoking history that could help us understand the link between smoking and PC. Life course smoking patterns capitalize on changes in smoking behaviors, avoiding the assumption of constant smoking that underlies the average smoking index and smoking status at interview.

Regarding PC aggressiveness, our findings are consistent with those reported by Islami, et al.*,* [2]. They evaluated the impact of PSA screening on smoking and PC risk, and found a positive association between former (RR: 1.08; 95% CI, 1.01–1.16) as well as ever (RR: 1.06; 95% CI, 1.00–1.12) smokers and PC incidence, but only among studies completed before the PSA screening era (1995 or earlier). In contrast to developed countries where higher PC incidence occurs at the expense of well differentiated PC or Gleason ≤6 [30], Mexico has no PC population-screening program and most PC cases are classified as aggressive PC (Gleason ≥7) at the time of diagnosis. The aggressive PC (Gleason ≥7) frequency observed in this study (73%) is similar to that reported by Gomez-Guerra et al., [15], in a population-based study, where most of the PC prevalent cases (92.3%) had been considered high-grade or aggressive cancer at diagnosis.

Some limitations of our study must be mentioned. Case-control studies are prone to selection bias due to differential participation rates between cases and controls. However, we believe that the possibility of differential smoking patterns between participants and non-participants is low. Participation rates for cases and controls were similar, and we did not observe differences in relation to some sociodemographic characteristics (age at the interview, birthplace, marital status and educational level) according to their participation status [14]. Selection bias could also have occurred because controls failed to represent the base population from which the cases arose. However, in our study this possibility seems remote, since we used community-based selection of controls and the prevalence of smoking behaviors among our controls were similar to those reported in several recent national surveys. The prevalence of ever smokers reported by the National Health and Nutrition Survey [31] for males over 60 years old and residents of Mexico City (65.6 vs. 67.2%) was similar to that observed among our controls. There is no information available about the distribution of smoking patterns A and B in the general population. Nevertheless, available data suggest that most of ever-smoker Mexican males (76.4%) in urban areas have a low smoking intensity habit (< 11 cigarettes/day) [32]. This figure is consistent with our results, where most of the smoking controls (88.9%) experienced a life-course Pattern A, characterized by a median of 4 cigarettes/day and 17 years old at smoking onset; only a small fraction of smoking controls (11.1%) were characterized as smoking pattern B, with a median of 17.9 cigarettes/day.

We had to rely on the memory of cases and controls to reconstruct their smoking history, and recall bias is a possibility. This seems unlikely, since a previous US study [33] analyzed the recall bias potential for ever smoker in cases with prostate cancer and controls, and found similar and moderate levels of agreement between medical records and self-reported questionnaires. Depending on age (40 to 64 years and 65 to 69 years), among cases Kappa ranged between 0.58 and 0.68, while among controls it ranged from 0.62 to 0.66. Still, future studies should aim to capture smoking status from medical records, to directly estimate the bias potential. Finally, we estimated that our study did not have enough power to detect a difference between smokers and non-smokers smaller or equal to 1.5; most previous studies regarding PC and smoking status have reported associations ≤1.5.

Multivariate models were adjusted for known potential confounders; however, residual confounding due to alcohol consumption remains. Alcohol intake was evaluated within a 3-year timeframe before the interview for controls or diagnosis for cases; alcohol consumption was low and similar between cases and controls (one glass/week for cases vs. <one drink/week for controls; *p* = 0.9) and adjustment by alcohol consumption did not modify the observed association. The association between PC and alcohol consumption is controversial [34], but a recent meta-analysis suggests that alcohol consumption could increase PC risk by less than 10% [35]. Another potential confounder is body mass index (BMI), however, we did not include this variable in the final models. BMI was measured at interview and we decided not to include it because it could be affected by disease progression and misrepresent BMI at time of disease onset.

## Conclusions

Our results suggest that the association between smoking and PC is complex, and could depend on changes in smoking intensity over the life course. The prevalence of smoking pattern B among controls living in Mexico City was 7.4%; assuming that controls represent the general male population, approximately 9% of poorly differentiated PC (Gleason ≥8) could be attributable to life course smoking pattern B. Inconsistencies observed in previous studies could respond to disregard for PC aggressiveness and limitations of exposure assessment methods. Using smoking patterns throughout life course could help us understand the link between smoking and PC, but further validation of the method is needed, particularly in the context of prospective studies with different smoking prevalence and intensity than ours.

## Additional files

Additional file 1: Smoking Questions used in this study. Details of the complete sequence of smoking question used for classifying smokers subjects. (DOCX 55 kb)

Additional file 2: Supplementary Information. Methodology for calculation of life course smoking patterns. Details of the complete command sequence used for estimating the smoking patterns. (DOCX 35 kb)

Additional file 3: Average smoking index (pack/year) at each different stage between life course smoking patterns. Smoking intensity variation (average smoking index at each different stage) in each identified smoking patterns. (DOCX 16 kb)

Additional file 4: Comparison between traditional smoking assessment approaches and life course smoking patterns among controls. Details about the potential misclassification bias attributable to use of traditional smoking assessment approaches. (DOCX 19 kb)

## Abbreviations

95% CI: 95% Confidence Interval
BMI: Body mass index
OR: Odds ratio
PA: Physical activity
PAHs: Polycyclic aromatic hydrocarbons
PC: Prostate cancer
PSA: Prostate Specific Antigen
