# Supplementary material for: Smoking and prostate cancer: a life course analysis
Source: BMC Cancer. 2018 Feb 7;18:160. doi: 10.1186/s12885-018-4065-7 (PMC5803914; doi:10.1186/s12885-018-4065-7)
Supplement: Supplementary file 1 — Smoking Questions used in this study. Details of the complete sequence of smoking question used for classifying smokers subjects. (DOCX 55 kb) [file 12885_2018_4065_MOESM1_ESM.docx]

**Additional file 1: Smoking Questions used in this study**

1. **Have you ever smoked at least 100 cigarettes along your life?**

*Smoking duration for each period was estimate taking into account age at start and at quit smoking

No

*Never Smoker*

Yes

*Ever Smoker*

Yes

*Current Smoker*

**2.- Do you currently smoke?**

No

*Former Smoker*

**4.-At what age did you quit smoking?**

**(B)**

**3.- At what age did you start smoking?**

**(A)**

**Total smoking duration= B-A**

**or**

**Current age-A**

Smoking duration

(years)*

Average number of cigarettes during each period

Smoking Index

**At each period**

**Smoking Intensity:**

**5.-On average, how many cigarettes did you usually or habitually smoke at:**

5a.- ≤ 20 years old

5b.- 21-30 years old

5c.- ≥ 31 years old

C

D

E

(C x F)/20

(D x G)/20

(E x H)/20

F

G

H

Cumulative lifespan smoking index= (total average number of cigarettes x total smoking duration)/20

**Abstinence time =Age at interview-B**
