# Supplementary material for: Smoking and prostate cancer: a life course analysis
Source: BMC Cancer. 2018 Feb 7;18:160. doi: 10.1186/s12885-018-4065-7 (PMC5803914; doi:10.1186/s12885-018-4065-7)
Supplement: Supplementary file 2 — Supplementary Information. Methodology for calculation of life course smoking patterns. Details of the complete command sequence used for estimating the smoking patterns. (DOCX 35 kb) [file 12885_2018_4065_MOESM2_ESM.docx]

**Additional file 2. Methodology for calculation of life course smoking patterns**

For the estimation of life course smoking patterns, first we included the smoking indexes from each life stage into a .csv file, and then we set the route to the file and read it. We verified the dataset, afterward we installed and called the kml packages. We identified the variable-trajectory and specified the number of pattern for the drawing of the plot, which we could adjust depending on observation. All the patterns accomplished the quality criteria of Calinski & Harabasz, also we compared the patterns obtained by a different method such as k-means++. The complete command sequence used was:

setwd("~/Desktop/Tobacco/Tobacco patterns")

datos<-read.csv("Tobacco.csv", header=TRUE, sep=",")

head(data)

install.packages("kml")

library(kml)

cldSDQ <- cld(data, timeInData = 2:4)

cldSDQ

kml(cldSDQ, nbClusters = 2, nbRedrawing = 20, toPlot = "both")

#kml(cldSDQ)

datos$clusters <- getClusters(cldSDQ, 2)

#epipageGroupAD <- epipageShort[epipageShort$clusters %in% c("A", "D"), ]

write.table(datos,file="Tobaccopatterns.csv", sep=",",col.names=TRUE)

For comparison between smoking patterns estimated by k-means+ and k-means++ method we used the following command sequence:

Tobaccopatternsb<-read.csv(file.choose(),header = TRUE)

View(Tobaccopatternsb)

Tobaccopatternsb.features=Tobaccopatternsb

Tobaccopatternsb.features$cluster<-NULL

View(Tobaccopatternsb.features)

results<-kmeans(Tobaccopatternsb.features,2)

results

results$size

results$cluster

table(Tobaccopatternsb $cluster,results$cluster)

#####Tridimensional graph

install.packages("rgl")

require(rgl)

install.packages("RColorBrewer")

library(RColorBrewer)

plot3d(Tobaccopatternsb.features$indix1, # x variable

Tobaccopatternsb.features$indix2, # z variable

Tobaccopatternsb.features$indix3, # y variable

xlab = "Indix1",

zlab = "Indix2",

ylab = "Indix3",

col=brewer.pal(4,"Set3")[unclass(Tobaccopatternsb $cluster)],size=8
