# Supplementary material for: Smoking and prostate cancer: a life course analysis
Source: BMC Cancer. 2018 Feb 7;18:160. doi: 10.1186/s12885-018-4065-7 (PMC5803914; doi:10.1186/s12885-018-4065-7)
Supplement: Supplementary file 3 — Average smoking index (pack/year) at each different stage between life course smoking patterns. Smoking intensity variation (average smoking index at each different stage) in each identified smoking patterns. (DOCX 16 kb) [file 12885_2018_4065_MOESM3_ESM.docx]

**Additional file 3. Average smoking index (pack/year) at each different stage between life course smoking patterns**

| Smoking pattern | | Average smoking index at each different stage (pack/year) | | |
| --- | --- | --- | --- | --- |
|  |  | ≤ 20 years old | 21-30 years | > 30 years old |
| Ever smokers | Pattern A | 0.93 ± 1.62 | 3.21 ± 3.20* | 5.92 ± 5.7* |
|  | % of change |  | 245% | 536% |
|  | Pattern B | 2.50 ± 2.80 | 10.24 ± 6.85* | 39.47 ± 20.89* |
|  | % of change |  | 310% | 1478% |
| Former smokers | Pattern A | 0.98 ± 1.66 | 3.21 ± 3.24* | 4.59 ± 4.97* |
|  | % of change |  | 227% | 368% |
|  | Pattern B | 2.73 ± 2.57 | 12.25 ± 7.76* | 35.43 ± 16.06* |
|  | % of change |  | 349% | 1198% |

*: ANOVA test p<0.01.
