# Supplementary material for: Smoking and prostate cancer: a life course analysis
Source: BMC Cancer. 2018 Feb 7;18:160. doi: 10.1186/s12885-018-4065-7 (PMC5803914; doi:10.1186/s12885-018-4065-7)
Supplement: Supplementary file 4 — Comparison between traditional smoking assessment approaches and life course smoking patterns among controls. Details about the potential misclassification bias attributable to use of traditional smoking assessment approaches. (DOCX 19 kb) [file 12885_2018_4065_MOESM4_ESM.docx]

**Additional file 4. Comparison between traditional smoking assessment approaches and life course smoking patterns among controls**

| **Traditional Smoking assessment approaches.** | **Life course smoking patterns among ever smoker^a^** | | | | | | | | | | | | | |
| --- | --- | --- | --- | --- | --- | --- | --- | --- | --- | --- | --- | --- | --- | --- |
|  | **Pattern A (n=474)** | | | | | | |  | **Pattern B (n=59)** | | | | | |
|  | n (%) | | Age at onset  Median  (Min-Max) |  | Number of cigarettes  Median  (Min-Max) |  | Duration  (Years)  Median  (Min-Max) |  | n (%) | Age at onset  Median  (Min-Max) |  | Number of cigarettes  Median  (Min-Max) |  | Duration  (Years)  Median  (Min-Max) |
| **Smoking status at interview^b^** | | | | | | | | | | | | | | |
| Former smoker>15a  Former smoker≤15a  Current Smoker | 197 (96.6)  82 (84.5)  195 (83.8) | 17 (7-55)  16 (11-40)  17 (8-53) | |  | 4 (1-30)  5 (1-20)  4 (0.7-40) |  | 21.5 (2-50)  40 (13-64)  47 (15-69) |  | 7 (3.4)  15 (15.5)  37(15.9) | 15 (6-16)  14 (12-18)  15.5 (9.25) |  | 23 (12-47)  17.5 (9-60)  14 (6.38) |  | 39 (21-45)  44 (20-56)  54 (37-74) |
| **Average smoking index in life (packs/year)** | | | |  |  |  |  |  |  |  |  |  |  |  |
| 0.15 - 5.2  5.3 – 14.0  14.0- 112.0 | 177 (100)  179 (100)  118 (66.7) | 18 (8-55)  17 (10-40)  15 (8-40) | |  | 2 (0.7-20)  4 (2-30)  9.1 (5-40) |  | 23 (2-59)  40 (6-69)  42 (9-63) |  | 0  0  59 (33.3) | 15(6-25) |  | 15.5 (6-60) |  | 48 (20-74) |

a Pattern A: characterized by males who reported low and constant smoking intensity and Pattern B: males with initial period of low smoking intensity, followed by an increase during the second period.

b Former smokers were categorized according to cessation time before interview
